# Surgical Repair Using Suture Bridge Technique for Triceps Tendon Avulsion

**DOI:** 10.1155/2021/5572126

**Published:** 2021-04-20

**Authors:** Ryogo Furuhata, Yusaku Kamata, Aki Kono, Taichi Nishimura, Shinya Otani, Hideo Morioka

**Affiliations:** Department of Orthopaedic Surgery, National Hospital Organization Tokyo Medical Center, 2-5-1, Higashigaoka, Meguro-ku, Tokyo, Japan

## Abstract

Triceps tendon avulsion is a rarely occurring tendinous injury. Various surgical procedures, such as repair using sutures through the transosseous tunnel or suture anchors, have been reported for treating triceps tendon avulsion. However, standard surgical treatment has not yet been established. Here, we present a case of triceps tendon avulsion treated using the suture bridge technique. A 58-year-old man who fell on his left elbow from standing height presented to our hospital. Plain radiography revealed an avulsion fracture of the left olecranon process, suggesting triceps tendon avulsion. We performed surgical repair of the avulsed bone fragments and ruptured triceps tendon. We inserted suture anchors into the ulna, proximal to the fracture site, and passed the sutures through the full thickness of the triceps. Subsequently, fracture fragments were reduced and fixed by pulling them together with the triceps. We inserted knotless anchors into the ulna distal to the fracture site and fixed the avulsed bone fragments and triceps tendon using the suture bridge technique. The patient recovered well in five months and reported no elbow pain or limited range of motion. This suture bridge technique is advantageous as it prevents iatrogenic fracture and knot irritation, and it would be indicated in cases with poor bone quality or thin skin soft tissue of the olecranon.

## 1. Introduction

Triceps tendon rupture occurs rarely, accounting for less than 0.8% of all upper limb tendon injuries [1]. The injury is thought to be caused by eccentric load on the triceps during a fall on an outstretched arm [2]. Since the site of rupture is most commonly located at osseous insertion, patients with triceps tendon rupture often present with avulsion fractures of the olecranon process [3].

Tears involving >50% of the tendon or complete ruptures are considered an indication for surgical treatment [2, 4]. To date, repair using sutures through transosseous tunnels or suture anchors has been predominantly reported; however, standard surgical treatment has not yet been established.

Here, we present a case of triceps tendon avulsion, wherein we used the suture bridge technique, which is often performed for avulsion fractures of the greater tuberosity.

## 2. Case Presentation

**Figure 1:**
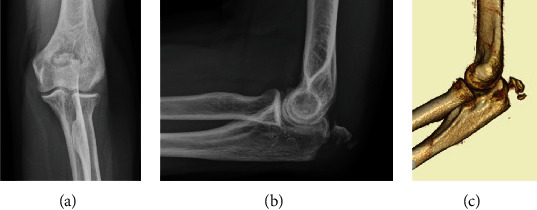
Plain radiography and computed tomography (CT) images of the left elbow. Plain radiography revealed an avulsion fracture of the olecranon process (a, b). CT shows small, avulsed fragments with comminuted bone (c).

A 58-year-old man who fell on his left elbow from a standing height presented to our hospital. The patient had a history of epilepsy that was well-controlled via pharmacotherapy. Physical examination demonstrated difficulty in moving the left elbow due to pain. There were no findings suggestive of nerve or vascular injury. Plain radiography revealed an avulsion fracture of the left olecranon process (Figures 1(a) and 1(b)). Computed tomography revealed small, avulsed fragments with comminuted bone (Figure 1(c)). These imaging findings suggested an avulsion fracture of the olecranon at the site of attachment of the triceps brachii tendon. Therefore, we performed an open reduction and internal fixation.

**Figure 2:**
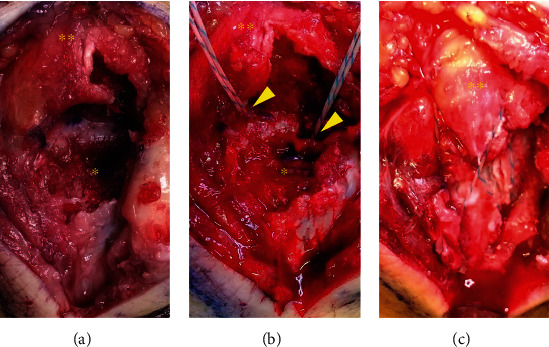
Intraoperative images of the left elbow. The triceps brachii tendon (double asterisk) is continuous with the fracture fragment (asterisk) (a). Two suture anchors (arrowheads) were inserted into the ulna proximal to the fracture site (b). After passing the suture thread through the triceps brachii tendon, the fracture fragments and triceps were reduced and fixed together using the suture bridge technique (c).

**Figure 3:**
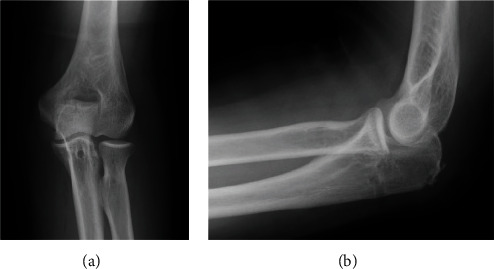
Plain radiography images of the left elbow after the osteosynthesis. Postoperative plain radiography images demonstrated that the avulsion fracture fragments were reduced (a, b).

The patient underwent general anesthesia and was placed in the right lateral decubitus position. A curvilinear posterior midline incision was made over the left olecranon (curved to the radial side over the olecranon to avoid direct incision over the tip of the bone). The avulsed bone fragments were dislocated and attached to the triceps brachii tendon. The fracture fragments were temporarily flipped with the triceps brachii, and the fracture site was freshened (Figure 2(a)). We inserted two JuggerKnot® anchors (Zimmer Biomet, Warsaw, IN, USA) into the ulna, proximal to the fracture site (Figure 2(b)). Sutures were passed through the full thickness of the triceps, and the dislocated bone fragments were reduced by pulling them together with the triceps brachii. With the elbow kept in the reduced position of 45° of flexion, we inserted two Quattro® Link Knotless anchors (Zimmer Biomet, Warsaw, IN, USA) into the ulna distal to the fracture site and fixed the dislocated bone fragments using the suture bridge technique (Figure 2(c)). Postoperative plain radiography demonstrated that the avulsion fracture fragments were reduced (Figure 3). After surgery, the elbow was fixed at 90° for 3 weeks prior to the start of the range of motion exercises. Two months after surgery, weight bearing was permitted. Five months after surgery, the patients had no elbow pain or limited range of motion (flexion 0°, extension 150°), and the Mayo Elbow Performance Score was 100.

## 3. Discussion

**Figure 4:**
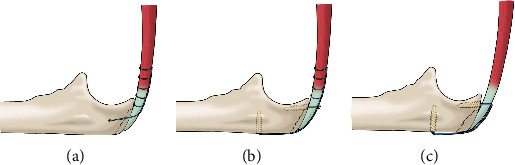
Schematic illustration for the repair of triceps tendon rupture or avulsion. The conventional transosseous technique involves passing nonabsorbable sutures placed in the triceps tendon through bone tunnels in the olecranon and tying them together over a bony bridge (a). In the suture anchor technique described by Yeh et al., the triceps tendon is repaired using four suture anchors and nonabsorbable sutures placed in the triceps tendon (Yeh et al., 2010) (b). Most of the reports about conventional transosseous or suture anchor technique have used the Krackow whipstitch technique for the triceps tendon (a, b). In our patient, the avulsed fragments and triceps tendon were reduced and fixed using the suture bridge technique after suture anchors were inserted proximal and distal to the ulnar fracture site (c).

To date, various surgical methods have been reported for the treatment of triceps tendon rupture or avulsion, each with its advantages and disadvantages. However, a standard surgical treatment method has not yet been established. The most common primary technique involves using transosseous tunnels and suture anchors. In this transosseous technique, the nonabsorbable sutures placed in the triceps tendon are passed through bone tunnels in the olecranon and tied together over a bony bridge [3] (Figure 4(a)). Recently, researchers have proposed techniques using suture anchors. Yeh et al. [4] reported that the double-row suture anchor technique using 4 anchors (Figure 4(b)) provided superior footprint contact and significantly less displacement during cyclic loading than traditional transosseous techniques and concluded that the double-row suture anchor technique could reconstruct the triceps brachii more anatomically. Furthermore, Clark et al. [5] proposed a hybrid transosseous suture anchor technique that combines transosseous tunnels and a knotless anchor. This technique also showed significantly higher load and cycle to failure and greater footprint coverage than the traditional transosseous technique [5, 6]. Contrary to these findings, however, a study comparing the clinical results of the transcutaneous technique and the suture anchor technique against triceps tendon rupture showed no significant differences in functional outcome and rerupture rate between the two groups [7].

In our case, we inserted suture anchors proximal and distal to the ulnar fracture site at the footprint of the triceps tendon, and the avulsed fragment and triceps tendon were reduced and fixed using the suture bridge technique (Figure 4(c)). The suture bridge technique for avulsion fractures is widely used for greater tuberosity fractures, as heavy sutures incorporating tendons are thought to provide better fixation and decrease the risk of the sutures being pulled through the comminuted or osteoporotic bone fragments [8]. In this case, four high-strength sutures from the anchor successfully clamped the olecranon fragment and triceps tendon on the surface.

Our method has three major clinical features. First, this method did not involve the risk of iatrogenic fracture during transcutaneous tunnel drilling, which can occur during conventional transcutaneous techniques. In addition, the suture bridge technique in this case helps avoid further fragmentation because the sutures are passed through the triceps, rather than fractured fragments. Second, in the previously reported double-row suture anchor technique, relatively large knots remain on the superior border of the triceps tendon. There have been concerns that this would result in postoperative knot failure or subcutaneous irritation [5, 6]. However, this issue was resolved using a knotless anchor in this method using the suture bridge technique. Third, most of the reports using conventional transosseous or suture anchor techniques use the Krackow whipstitch technique for the triceps tendon [3–6]; however, in our case, we did not perform this whipstitch technique. The increased amount of suture through the tendon may contribute to increased strength and durability; however, since the Krackow suture is a locking-type suture, it may impair the blood flow to the suture site [9].

In the present technique, careful attention is required to ensure that the suture anchors inserted proximally do not cause intra-articular perforation. To prevent intra-articular perforations, it is vital to use radioscopy while drilling to confirm that the drill does not perforate the articular surface. In addition, we used an anchor with traditional high-strength sutures. However, an anchor with a wide tape such as FiberTape® (Arthlex, Naples, FL) could have increased the compressive surface area and pull-out strength of the suture construct [10] and was considered an area requiring further improvement.

In summary, we present a surgical procedure for treating triceps tendon avulsion using the suture bridge technique. The procedure is beneficial especially for cases with poor bone quality or thin skin soft tissue of the olecranon because it may help prevent iatrogenic fracture and knot irritation.

## Data Availability

The data of the patients are in the clinical records of the hospital.

## References

[1] Anzel S. H., Covey K. W., Weiner A. D., Lipscomb P. R. (1959). Disruption of muscles and tendons; an analysis of 1,014 cases. *Surgery*.

[2] Bach B. R., Warren R. F., Wickiewicz T. L. (1987). Triceps rupture. *The American Journal of Sports Medicine*.

[3] van Riet R. P., Morrey B. F., Ho E., O'Driscoll S. W. (2003). Surgical treatment of distal triceps ruptures. *The Journal of Bone and Joint Surgery. American Volume*.

[4] Yeh P. C., Stephens K. T., Solovyova O. (2010). The distal triceps tendon footprint and a biomechanical analysis of 3 repair techniques. *The American Journal of Sports Medicine*.

[5] Clark J., Obopilwe E., Rizzi A. (2014). Distal triceps knotless anatomic footprint repair is superior to transosseous cruciate repair: a biomechanical comparison. *Arthroscopy*.

[6] Paci J. M., Clark J., Rizzi A. (2014). Distal triceps knotless anatomic footprint repair: a new technique. *Arthroscopy Techniques*.

[7] Horneff J. G., Aleem A., Nicholson T. (2017). Functional outcomes of distal triceps tendon repair comparing transosseous bone tunnels with suture anchor constructs. *Journal of Shoulder and Elbow Surgery*.

[8] Lee B. G., Cho N. S., Rhee Y. G. (2012). Modified Mason-Allen suture bridge technique: a new suture bridge technique with improved tissue holding by the modified Mason-Allen stitch. *Clinics in Orthopedic Surgery*.

[9] Dong Z., Qiu B., Pan Y., Wu S., Hong X., Liu F. (2018). Improved Krackow method combined with unilateral mattress suture for treating recent Achilles tendon rupture. *Journal of the College of Physicians and Surgeons–Pakistan*.

[10] Caldwell P. E., Evensen C. S., Vance N. G., Pearson S. E. (2018). Distal triceps speed bridge repair. *Arthroscopy Techniques*.

